# Artificial Intelligence-Assisted Invasion Risk Scoring in Tru-Cut Biopsies of Ductal Carcinoma in Situ; Predictive Role of Estrogen Receptor and Her2 Expression

**DOI:** 10.5146/tjpath.2026.14901

**Published:** 2026-09-15

**Authors:** Evren Uzun, Kübra Bulut

**Affiliations:** Department of Pathology, Gaziantep University Hospital, Gaziantep, Türkiye; Department of Pathology, Şanlıurfa Education and Research Hospital, Şanlıurfa, Türkiye

**Keywords:** Tru-cut biopsy, Breast, DCIS, Invasion, Artificial, Intelligence

## Abstract

Objective: Invasion can be detected in up to 50% of excision specimens in cases diagnosed with ductal carcinoma in-situ (DCIS) in tru-cut biopsies. The predicting of invasion is critical for managing the treatment. The objective of this study was to ascertain the predictive role of histological characteristics and estrogen receptor (ER) and HER2 expression of DCIS with respect to invasion and to create an artificial intelligence (AI)-assisted invasion risk scoring.

Material and Methods: A total of 140 tru-cut biopsies with DCIS were included. We evaluated the predominant structural pattern, nuclear grade, presence of comedonecrosis, and ER/HER2 expressions and their relationship with invasion upstage. Based on the results, an invasion risk scoring system was created using OpenAI-ChatGPT 5.2.

Results: Of the 140 cases, 70 (50%) were high-grade (DCIS-3), 49 (35%) were intermediate-grade (DCIS-2), and 21 (15%) were low-grade (DCIS-1). DCIS-1 and DCIS-2 showed significant association with a cribriform pattern, and DCIS-3 showed with a solid pattern. When the results were compared with those of the final specimens, invasion upstaging occurred in 9.5% of DCIS-1, 38.7% of DCIS-2, and 52.8% of DCIS-3 cases. A solid pattern, high nuclear grade, comedonecrosis, and ER-/HER2- profile showed high correlation with invasion. In AI-assisted risk scoring, DCIS cases were divided into three groups: low, medium, and high risk. The invasion upstaging rates in these groups were 11.44%, 28.97%, and 64.19%, respectively.

Conclusion: DCIS with high nuclear grade, comedonecrosis, a solid pattern, and the ER-/HER2+ or ER-/HER2- immunoprofile should be considered high risk for invasion. They should be approached as invasive tumors and evaluated in terms of lymph node staging and neoadjuvant treatment.

## INTRODUCTION

Ductal carcinoma in situ (DCIS) is acknowledged as a non-essential precursor lesion of invasive breast carcinoma (1). Despite the fact that DCIS is present in approximately 20% of all breast carcinomas, its diagnosis is becoming increasingly common as a result of the technological advancements such as mammography (2–4). Given that pure DCIS is theoretically devoid of metastatic potential, lymph node metastasis is not anticipated. However, invasion can be identified in the final specimen of a patient diagnosed with DCIS via tru-cut biopsy, with a reported incidence of 13-48% (5–8). Consequently, patients may require lymph node staging and supplementary surgical intervention. The identification of DCIS cases with a high probability of invasion, that is, the revelation of data that can predict invasion in cases diagnosed with DCIS in tru-cut biopsy, will facilitate the utilisation of techniques that may provide additional benefits in patient management, such as sentinel lymph node sampling. In the course of various studies conducted to date, three groups of factors that are thought to predict invasion in cases diagnosed with DCIS have been proposed. These factors have been clinically reported as age and lesion characteristics, radiologically as the number and type of biopsies, mammographic density, MRI findings, and histopathologically as high nuclear grade, HER2 positivity, comedonecrosis, and solid pattern (9–21). In this study, we investigated the predictive role of histologic and immunohistochemical features in determining invasion in cases diagnosed as DCIS in tru-cut biopsies from a novel perspective. Furthermore, we proposed a artificial intelligence (AI)-assisted invasion risk scoring system for DCIS cases, the first of its kind in the literature, which is similar to the system used in invasive breast carcinomas.

## Materials and Methods

### Patient Selection

A total of 140 patients were included in the study. The initial material obtained from all patients was a tru-cut biopsy. Those with final excisional specimens (mastectomy, lumpectomy, breast conservation, etc.) were prioritised. Patients with non-tru-cut biopsies, no excisional specimens, microinvasion/invasion or suspicion of microinvasion/invasion on tru-cut biopsy, lymphovascular or perineural invasion, previous breast diagnosis and Paget’s disease of the nipple were excluded from the study. The clinical and demographic data were obtained from the patient records.

### Histopathological Evaluation

The histopathological evaluation comprised a re-evaluation of the tru-cut biopsies of DCIS by two pathologists with experience in breast pathology. This entailed an assessment of the predominant growth pattern (solid, cribriform, micropapillary, papillary, flat), the presence or absence of comedonecrosis and nuclear grade, and the final specimens for invasion.

### Immunohistochemical Analysis

A total of 140 cases were evaluated for estrogen receptor (ER) (Ventana SP1 clone) and HER2 (clone 4B5; Ventana Medical Systems, Inc.) according to the evaluation criteria specified in the fifth edition of the World Health Organization (WHO) classification of breast tumours (22). In cases where the HER2 result was deemed to be equivocal, a confirmation test was conducted using the Ventana HER2 Dual in situ hybridization (ISH) DNA probe cocktail, in accordance with the following criteria.

### Evaluation criteria;

ER; Staining at any intensity of 1% or more

HER2;

**3+**: Circumferential membrane staining that is complete, intense, and in > 10% of tumor cells

**2+**: Weak to moderate complete membrane staining observed in > 10% of tumor cells

**1+**: Incomplete membrane staining that is faint / barely perceptible and in > 10% of tumor cells

**0**: No staining is observed, or incomplete membrane staining that is faint / barely perceptible and in ≤ 10% of tumor cells

DCIS cases in tru-cut specimens were evaluated in four groups as follows: hormone positive (ER+/HER2-), HER2 positive (ER-/HER2+), ER-/HER2-, and ER+/HER2+. This classification system is consistent with the immunoprofile groups of invasive breast carcinoma specified in the fifth edition of the World Health Organization’s Tumours (22).

### AI-assisted Invasion Risk Scoring

AI-assisted model construction;

A file containing the raw histopathological and immunohistochemical data for 140 cases, anonymised and individually recorded, was uploaded to the ChatGPT 5.2 database. The AI tool was instructed as follows: ‘The data for 140 cases diagnosed with ductal carcinoma in situ on Tru-cut biopsy are available in the relevant file. By analyzing these data and comparing them with similar studies in the literature, would you create an invasion risk scoring system similar to the Nottingham scoring system used in invasive breast carcinomas?’ The request was made to evaluate ER and HER2 status together, similar to the subtyping used in invasive breast carcinomas.

The following results were obtained from the command:

The construction of a LASSO (least absolute shrinkage and selection operator) logistic regression model, with the assistance of artificial intelligence, was undertaken for the purpose of predicting invasion upstaging.

The following steps were taken in the creation of the model: Automated variable selection was performed. The penalisation of overfitting was maintained. The interpretability of the model was deemed suitable for clinical and pathological applications.

The model incorporated the following variables:

A comprehensive consideration was given to the following factors: the presence of nuclear grade, the presence of a histological DCIS pattern, the ER status, the HER2 status, and the presence of comedonecrosis. The binary outcome variable was invasion upstaging (0 = no invasion, 1 = invasion present).

Model training and prediction;

The model was trained using the entire dataset to estimate regression coefficients and generate an individual predicted probability of invasion for each case. No imputation was performed, as complete data were available for all included variables.

Risk Stratification;

Initially, the variables were assigned individual scores based on their relationship with invasion upstaging (Table 1). The cases were stratified into three invasion risk categories based on the AI-derived predicted probabilities. The risk level is categorised as follows: low (total score 3-5), intermediate (total score 6-8), or high (total score 9-11). The risk group cutoff values were determined using tertile-based probability thresholds. This approach offers an objective, distribution-driven classification that does not rely on the utilisation of arbitrary thresholds.

**Table 1:** AI-assisted risk scoring of potential invasion

	**Dominant Pattern**	**Nuclear Grade**	**Comedonecrosis**	**Immunprofile**
**Score**	Solid and Flat (3)	High (3)	Yes (1)	ER-/HER2- (4)
	Cribriform (2)	Intermediate (2)	None (0)	ER-/HER2+ (3)
	Micropapillary (1)	Low (1)		ER+/HER2+ (2)
	Papillary (1)			ER+/HER2- (1)

**ER:** Estrogen receptor, **HER2:** Human epidermal growth factor receptor-2

Model Performance Evaluation;

The performance of the model was evaluated through the implementation of receiver operating characteristic (ROC) curve analysis. The area under the curve (AUC) was calculated as a measure of discriminative performance. The area under the curve (AUC) value was calculated as 0.88 by artificial intelligence, and this value was considered to be quite strong for the distinction between invasive and non-invasive cases.

### Statistical Analysis

Machine learning procedures were performed using Python-based analytical tools. The descriptive statistics of the data obtained from the study will be presented for numerical variables, including the mean, standard deviation, frequency, and percentage analysis for categorical variables. The conformity of numerical variables to a normal distribution will be examined using the Shapiro-Wilk test. The independent samples t-test/Mann-Whitney U-test will be employed for the comparison of numerical variables according to categorical variables, including two groups. The Analysis of Variance/Kruskal-Wallis test will be used for the comparison of numerical variables according to three or more categorical variables. Moreover, differences between categorical variables will be evaluated through a Chi-square analysis. Furthermore, the relationships between numerical variables will be examined through the use of Pearson correlation analysis or Spearman correlation analysis. The analyses will be conducted using the SPSS 22.0 software. The level of significance will be set at p < 0.05.

## RESULTS

The age range was 23-80 years, with a mean of 48 years. All cases were of the female gender. In tru-cut biopsies, 70 cases (50%) were diagnosed as high-grade ductal carcinoma in situ (DCIS nuclear grade 3/DCIS-3), 49 (35%) as intermediate-grade DCIS (DCIS nuclear grade 2/DCIS-2), and 21 (15%) as low-grade DCIS (DCIS nuclear grade 1/DCIS-1).

The cribriform pattern was the most prevalent among the DCIS-1 and DCIS-2 groups, accounting for 14/21(66.6%) and 24/49 (48.9%) cases, respectively. Conversely, the solid pattern was the most prevalent among the DCIS-3 group, comprising 48/70 (68.5%) cases. A significant correlation was observed between the solid pattern and high nuclear grade (p < 0.05).

Comedonecrosis was identified in a total of 54 DCIS cases, with a prevalence of 4.7% in the DCIS-1 group, 8.1% in the DCIS-2 group, and 70% in the DCIS-3 group. The presence of comedonecrosis was found to be significantly associated with high nuclear grade and the solid pattern (p < 0.05).

Upon immunohistochemical evaluation, 20 of the 21 cases in the DCIS-1 group were identified as ER+/HER2-, while one case exhibited ER-/HER2+ status. Of the 49 cases in the DCIS-2 group, 36 exhibited an ER+/HER2- immune profile, 7 demonstrated a ER-/HER2+ profile, and 6 displayed an ER-/HER2- profile. In the DCIS-3 group, 19 of the 70 patients were ER+/HER2-, 38 were ER-/HER2+, 2 were ER+/HER2+, and 11 were ER-/HER2-. The DCIS-1 and DCIS-2 groups exhibited a significant hormone-positive immune profile, while the DCIS-3 group demonstrated a significant ER-/HER2+ immune profile (p < 0.05). The ER-/HER2- cases were observed in the DCIS-2 and DCIS-3 groups, while the ER+/HER2+ cases were observed in the DCIS-3 group. However, no significant differences were identified between the groups. A summary of the histopathologic and immunohistochemical data for all three groups is provided (Table 2).

**Table 2:** Histopathologic and immunohistochemical characteristics of ductal carcinoma in situ cases

	**Comedonecrosis**	**ER+/HER2-**	**ER+/HER2+**	**ER-/HER2+**	**ER-/HER2-**	**Upstage to Invasion**
**DCIS 1 (n=21)**	**1 (4.7%)**	**20 (95%)**	**0**	**1 (4.7%)**	**0**	**2 (9.5%)**
Cribriform (n=14)	0	14	0	0	0	0
Solid (n=6)	1	5	0	1	0	2
Micropapillary (n=1)	0	1	0	0	0	0
**DCIS 2 (n=49)**	**4 (8%)**	**36 (73%)**	**0**	**7 (14%)**	**6 (12%)**	**19 (38.7%)**
Cribriform (n=24)	1	18	0	3	3	8
Solid (n=16)	3	10	0	3	3	9
Micropapillary (n=5)	0	4	0	1	0	1
Papillary (n=4)	0	4	0	0	0	1
**DCIS 3 (n=70)**	**49 (70%)**	**19 (27%)**	**0**	**40 (57%)**	**11 (15%)**	**37 (52.8%)**
Cribriform (n=16)	6	10	0	3	3	6
Solid (n=48)	40	6	2	34	6	29
Micropapillary (n=4)	2	2	0	1	1	1
Flat (n=2)	1	1	0	0	1	1
**Total (n=140)**	**54 (38.5%)**	**75 (53.5%)**	**2 (1.4%)**	**46 (32.8%)**	**17 (12%)**	**58 (41.4%)**

**DCIS: **Ductal carcinoma in situ, **ER:** Estrogen receptor, **HER2:** Human epidermal growth factor receptor-2, **n:** Number

**Figure 1:**
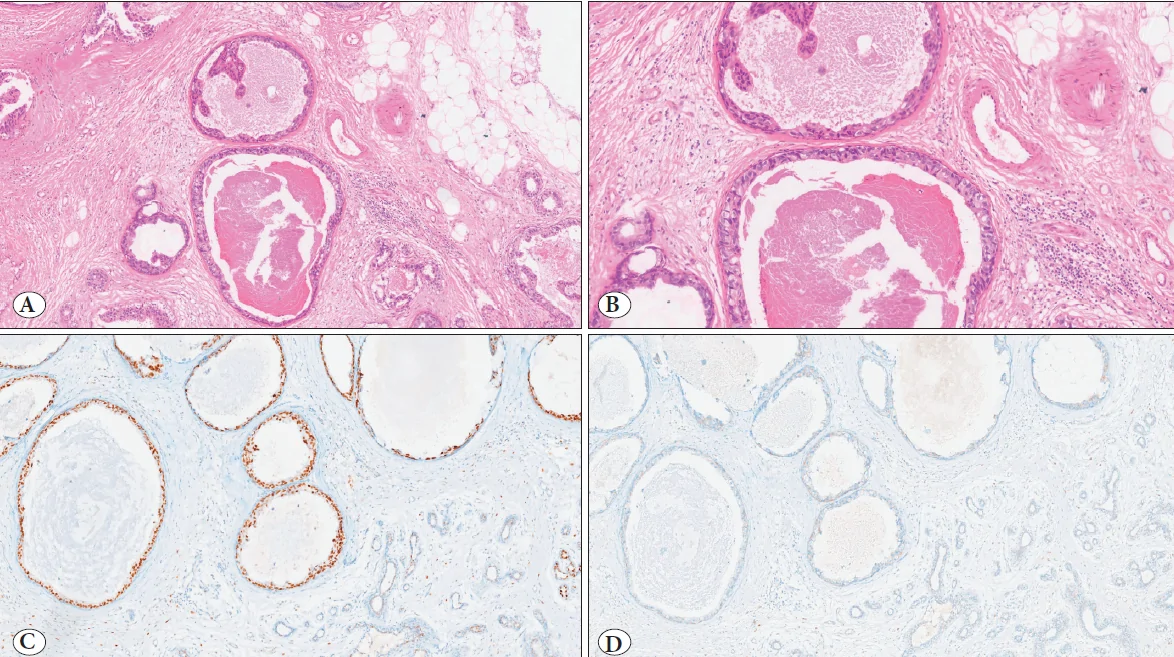
Ductal carcinoma in situ with flat pattern. In the mastectomy specimen of this case, invasive breast carcinoma with an ER+/ HER2- immunoprofile accompanying ductal carcinoma in situ was detected. A) Comedonecrosis, B) Grade 3 nucleus, C) Estrogen receptor positivity, D) HER2 negativity (A:20X, B:40X; H&E--C: ER antibody, 20X; E: HER2 antibody, 20X).

**Figure 2:**
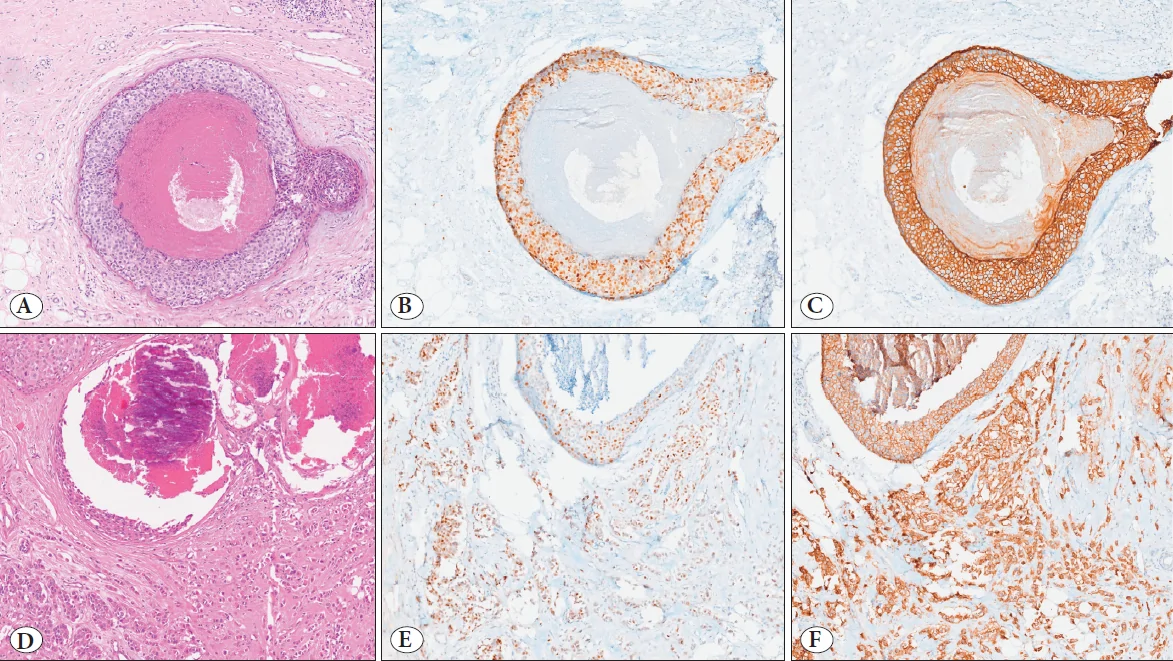
Tru-cut (A-C) and mastectomy (D-F) specimens from the same case. A) Ductal carcinoma in situ with solid pattern and comedonecrosis, B) ER positivity, C) HER2 positivity, D) Invasive breast carcinoma accompanied by ductal carcinoma in situ, E) Estrogen receptor positivity in both the in situ and invasive carcinoma, F) HER2 positivity in both the in situ and invasive carcinoma. (A&D: H&E; B&E: ER antibody; C&F: HER2 antibody; 20X).

The final specimens were available in all cases. Invasion was identified in the final specimens of 58 (41.4%) cases of ductal carcinoma in situ (DCIS). When the data were divided according to nuclear grade, 9% (2/21) of DCIS-1 cases, 38.7% (19/49) of DCIS-2 cases, and 52.8% (37/70) of DCIS-3 cases exhibited upstaging to invasion. There was a statistically significant increase in the rate of invasion from DCIS-1 to DCIS-3 (p < 0.05). In the initial biopsies of cases in which invasion was upstaged, 40 cases exhibited a solid histologic pattern, 14 cases exhibited a cribriform pattern, two cases exhibited a micropapillary pattern, and one case each exhibited a flat and papillary dominant pattern (Figure 1). The invasion rate was 57% (40/70) for all solid DCIS cases, 25.9% (14/54) for cribriform DCIS cases, and 25% (2/10, 1/2 and 1/4) for micropapillary DCIS, flat DCIS, and papillary DCIS cases. The rate of invasion in the final specimens of patients diagnosed with solid DCIS was significantly higher than in those with other patterns (p < 0.05) (Figure 2). Invasion was identified in the final specimens of 48 (88.8%) of the 54 DCIS cases with comedonecrosis. The rate was 11.6% in those without comedonecrosis (10/86). The presence of comedonecrosis was found to be a significant predictor of upstaging in DCIS cases (p < 0.05).

**Figure 3:**
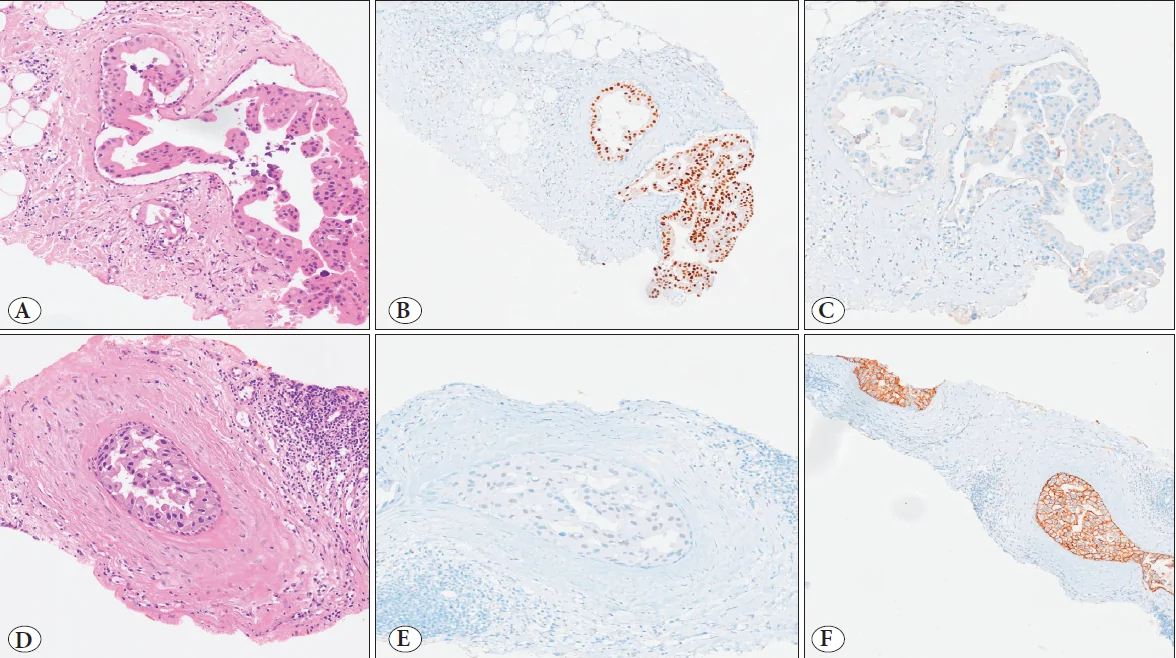
Cases of ductal carcinoma in situ are shown with (D-F) and without (A-C) invasion in the final specimens. These cases reflect the association of HER2 with invasion in cases with the same nuclear grade and without comedonecrosis. A) Micropapillary ductal carcinoma in situ with nuclear grade 2 and an absence of comedonecrosis, B) ER positivity, C) HER2 negativity, D) Cribriform ductal carcinoma in situ with nuclear grade 2, E) Estrogen receptor negativity, F) HER2 positivity. (A&D: 40X, H&E; B&E: ER antibody, 20X & 40X; C&F: HER2 antibody, 40X & 20X).

The relationship between the immune profile of DCIS cases and invasion is summarised in (Table 3). The results indicated that ER-/HER2- and ER-/HER2+ DCIS cases exhibited a markedly elevated risk of invasion in comparison to other cases (p < 0.05). When the two results were evaluated together, it was evident that ER negativity was a significant risk factor for invasion. Invasion was observed in one of two ER+/HER2+ cases, but the sample size was insufficient for statistical analysis (Figure 3). Upon analysis of the hormone pattern relationship, it was observed that the ER-/HER2+ profile-solid pattern exhibited a significantly higher association with invasion (p < 0.05), while the other immune profile pattern associations were not statistically significant (p > 0.05).

**Table 3:** Invasion relationship of immune and histologic features of DCIS cases

**Immune profile of DCIS**	**Number of cases**	**Upstage to Invasion**	**Dominant pattern of cases upstaging to invasion**				
			**Cribriform**	**Solid**	**Micropapillary**	**Papillary**	**Flat**
ER+/HER2-	75	14 (18.6%)	5	7	0	1	1
ER+/HER2+	2	1 (50%)	0	1	0	0	0
ER-/HER2+	46	28 (60.8%)	3	24	1	0	0
ER-/HER2-	17	15 (88.2%)	6	8	1	0	0

**DCIS: **Ductal carcinoma in situ, **ER:** Estrogen receptor, **HER2:** Human epidermal growth factor receptor-2

In conclusion, the statistical analysis revealed that the solid pattern, high nuclear grade, presence of comedonecrosis, and the ER-/HER2- immune profile were significant predictors of invasion in DCIS cases. Based on these results and the findings of similar studies in the literature, an AI-assisted invasion risk scoring system was developed.

A total risk score was calculated for each case according to the scoring system described in the material and method. The invasion rates and risk groups were summarised in (Table 4).

**Table 4:** Risk scoring for invasion upstage

**DCIS total risk score**	**Number of cases (n=140)**	**Invasion upstaging**	**Invasion upstaging rate**	**Average invasion rate**	
3	16	1	6.3%	11.44%	Low risk
4	9	1	11.1%		
5	10	2	20%		
6	12	2	16.7%	28.97%	Medium risk
7	12	3	25%		
8	14	6	42.9%		
9	15	9	60%	64.19%	High risk
10	38	24	63.2%		
11	14	10	71.4%		

**DCIS: **Ductal carcinoma in situ

## DISCUSSION

Tru-cut biopsy represents a reliable method for the diagnosis of the majority of breast lesions. The diagnosis of fibroepithelial lesions (fibroadenoma vs. phylloides tumour) (23,24) and papillary lesions can prove challenging in tru-cut specimens (25,26). Furthermore, the diagnosis of pure DCIS on tru-cut biopsy may not be representative of the entire lesion. Additionally, the inability to exclude invasion is also reported as one of the limitations of tru-cut biopsy. DCIS, although a non-invasive precursor lesion of invasive breast cancer, is theoretically considered not to metastasize to lymph nodes. Consequently, sentinel lymph node or axillary lymph node sampling is not routinely recommended in the absence of clinical indications (12,27–31). Nevertheless, numerous studies have documented an upstage to invasive tumour in up to 50% of final specimens from patients diagnosed with DCIS on tru-cut biopsy. In our study, the rate was found to be 41.4%. Although this result is consistent with the existing literature, it represents a very high invasion rate and demonstrates that the diagnosis of invasive tumour is delayed in approximately half of the cases, resulting in patients being deprived of lymph node staging. Consequently, numerous studies have been conducted in the literature to ascertain which cases diagnosed as DCIS on biopsy are at high risk of upstaging to invasive tumour and to identify the predictive radiological, histopathological, and immunohistochemical factors. The distinctive aspect of this study is that it primarily considers the immune profile and proposes an AI-assisted invasion risk calculation in accordance with the results.

Upon analysis of the histomorphological and immunohistochemical features of the DCIS groups, it was observed that the DCIS-1 and DCIS-2 groups exhibited a cribriform predominant pattern and an ER+/HER2- profile with greater frequency, while the DCIS-3 cases demonstrated a predominant solid pattern and an ER-/HER2+ profile. Comedonecrosis was found to be correlated with high nuclear grade, a finding that was consistent with the existing literature (1).

The analysis revealed no statistically significant correlation between age and the presence of invasion in the final specimen. This finding aligns with the conclusions drawn in the existing literature (10). In our study, the histologically solid predominant pattern was found to be generally associated with higher nuclear grade and more frequent invasion compared to other patterns. Furthermore, the solid pattern was observed to be associated with a higher incidence of comedonecrosis and HER2 positivity. While studies in the literature do not consistently identify a clear pattern, some studies have reported that the solid pattern is more frequently upstaged to invasion. However, other studies have indicated that the cribriform (13,32) papillary (9,32), and micropapillary (11) patterns are more frequently upstaged to invasion compared to other patterns. Conversely, some studies have indicated that there is no statistically significant correlation between histologic subtype and the detection of invasion in the final specimen (10,20,33). In studies examining the relationship between DCIS and invasion, the most frequently reported predictive factors for invasion are high nuclear grade and comedonecrosis. In our study, 9%, 38.7% and 52.8% of cases with low, intermediate and high nuclear grade, respectively, were found to have invasion in the final specimens. Furthermore, 88.8% of cases with comedonecrosis and 11.6% of cases without, invasion upstaging were identified. The upstaging of invasion in the final specimens of all five cases with comedonecrosis in the DCIS-1 and DCIS-2 groups indicates that comedonecrosis is a predictive factor irrespective of nuclear grade. It has been demonstrated that comedonecrosis may serve as a predictor of tumour recurrence and invasion (11). It is notable that a number of publications have also reported that nuclear grade and comedonecrosis are not predictive factors. In addition to the aforementioned data, there are several publications on lobular carcinogenesis, tumour size, periductal lymphocytic infiltrate, and calcification that address the predictive value of these factors. However, as these issues fall outside the scope of the present study, they will not be discussed further here.

In light of the predictive value of histomorphological data with lymph node involvement in cases where there is an upstage to invasion in the final specimens of cases diagnosed with ductal carcinoma in situ (DCIS) in tru-cut specimens, it was previously reported that only comedonecrosis may be predictive for lymph node involvement (22). However, lymph node involvement was excluded from the study since all cases included in this study did not have lymph node sampling.

The ER-/HER2- immunoprofile was identified as a predictive factor for invasion in the course of our study. Furthermore, cases classified as ER-/HER2+ exhibited a statistically significant increase in invasion upstaging compared to those classified as ER+/HER2-. In analogous studies in the literature, it was reported that ER-/PR- DCIS was more prone to invasion; however, statistical significance was not found (17). In another study, it was reported that an ER-/HER2+ profile was associated with a significant risk of invasion (13). In contrast to the aforementioned studies, other research has indicated that the hormone profile is not a significant predictor of invasion.

In addition to the findings reported in the literature, our study identified a solid pattern and an ER-/HER2- immunoprofile as new predictive factors for invasion.

In the existing literature, risk classifications in DCIS cases are typically based on radiological data. In one study, a risk algorithm was developed by scoring histologically cribriform pattern, medium-high nuclear grade, and hormone negativity, as well as size. HER2 was not included in the scoring system. In the present study, a comprehensive pathological AI-assisted risk classification was devised, incorporating ER and HER2 status, as well as histopathological pattern, comedonecrosis and nuclear grade as criteria. To the best of our knowledge, this is the first study to employ pathologic risk scoring in terms of invasion in DCIS cases. The relatively low number of cases included in the risk scoring was identified as a limitation of the study. A further limitation of this study is that the proposed AI-based invasion risk scoring system has not yet been validated in an independent external cohort. The establishment of the algorithm’s reproducibility, calibration, and real-world clinical applicability necessitates external validation in independent series, particularly those comprising matched tru-cut biopsy and resection specimens from different centres. This is due to the model’s development being undertaken using a single-centre retrospective series. It is imperative to acknowledge that this model should be regarded as a preliminary derivation model rather than a final decision-making tool for clinical practice.

## CONCLUSION

It is important to emphasize that the novelty of the present study does not lie in the identification of new individual risk factors for invasion in DCIS. High nuclear grade and comedonecrosis are well-established predictors and have been consistently reported in the literature. Rather, the originality of our approach resides in the systematic integration and quantitative weighting of conventional histopathological parameters together with ER/HER2 immunoprofile, resulting in a reproducible and pathology-based invasion risk scoring model. When high nuclear grade, comedonecrosis, solid pattern and ER-/HER2+ or ER-/HER2- immunoprofile are detected in tru-cut specimens, the patient should be evaluated for possible invasive tumour and lymph node involvement, with rates ranging from 13 to 48%. By providing an objective, pathology-driven risk estimate, the model may support surgeons in identifying patients who could benefit from SLNB at the time of initial surgery, particularly in borderline or controversial cases. Moreover, the standardized nature of the scoring system may facilitate multidisciplinary communication and help reduce variability in patient management across institutions. 

## Conflict of Interest

The authors declared that they have no conflict of interest.

## Ethical Approval

Approval was obtained from the Ethics Committee of Gaziantep

University for the study.
